# Surface-Imprinted Acrylamide Polymer-Based Reduced Graphene–Gold Sensor in Rapid and Sensitive Electrochemical Determination of αB-Conotoxin

**DOI:** 10.3390/s25051408

**Published:** 2025-02-26

**Authors:** Jia Cao, Jiayue Li, Tianyang Yu, Fei Wang

**Affiliations:** Department of Chemistry, School of Science, China Pharmaceutical University, Nanjing 211198, China; nanj9527@163.com (J.C.); cx841204@163.com (J.L.); wilion@sina.com (T.Y.)

**Keywords:** molecularly imprinted polymer, electrochemical sensor, conotoxins, acrylamide, graphene oxide–gold

## Abstract

The quantitative determination of conotoxins has great potential in the development of natural marine peptide pharmaceuticals. Considering the time-consuming sample pretreatment and expensive equipment in MS or LC-MS/MS analysis, an electrochemical sensor combined with molecularly imprinted polymer (MIP) is fabricated for the rapid monitoring of conotoxin αB-VxXXIVA to promote its pharmaceutical value and eliminate the risk of human poisoning. Electrochemically reduced graphene oxide–gold composite (rGO-Au) is modified with chitosan (CS) and glutaraldehyde (GA) to immobilize the macromolecular peptide, conotoxin αB-VxXXIVA. Subsequently, acrylamide (AAM) with a cross-linking agent, N,N′-methylene-bisacrylamide (NNMBA), is introduced into the rGO-Au electrode to form MIPs by electro-polymerization. The proposed MIP-based electrochemical sensor, PAM/αB-CTX/CS-GA/rGO-Au/SPE, exhibits satisfactory sensing performance in the detection of αB-VxXXIVA. Based on current change versus logarithm concentration, a wide linear range from 0.1 to 10,000 ng/mL and a low detection limit (LOD) of 0.014 ng/mL for this sensor are obtained. This work provides a promising method in electrochemical determination combined with MIP for the determination of macromolecular peptides.

## 1. Introduction

Conotoxins (CTXs) are kinds of peptide biotoxins originating from Conus venoms to offensively paralyze and shock the prey. Contrarily, these conopeptides are excellent pharmacological probes and drug leads for their affinity for multifarious ion channels and receptors [1,2]. There are over 10,000 reported sequences of these disulfide-rich peptides, and the majority of them present a highly conserved cysteine framework [3]. According to the similarities in their consensus signal sequence, conotoxins are classified by frameworks, and each framework is subdivided based on the pharmacological superfamilies [4,5]. CTXs from α-superfamilies such as α-CTX MI, α-CTX GI, α-CTX SI and α-CTX II, were recorded to possess binding affinity with different nicotinic acetylcholine receptor (nAChR) subtypes, including α3β4, α3β2, and α7 [6], which indicates that α-CTXs can distinguish certain nAChR subtypes. αB-VxXXIVA, a conotoxin characterized from Conus vexillum and subjected to αB3-superfamily and framework XXIV, was reported that could specifically combine with α9α10 nAChR in low micromolar affinity [7]. α9α10 nAChR, mainly presented in keratinocytes, is an important target for various analgesics and cancer chemotherapeutics [8,9]. αB-VxXXIVA acts as an antagonist of nAChR and exhibits great potency at the α9α10 subtype [10], which shows potential in promoting the development of novel therapeutics for cancer. However, the excess concentration of conotoxins could also cause poisoning, such as shock, paralysis, and even death (the lethal dose of α-CTX GI for mice is 20 nmol/g) [3]. In order to stimulate pharmaceutical value and eliminate side effects, an exclusive, sensitive and convenient method for the quantitative detection of the proposed peptide is indispensable. The reported methods for monitoring conotoxins include MS [11], LC-MS/MS [12] and immunoassay [13,14]. Considering the time-consuming pretreatment of samples and the high-cost equipment, MS or LC-MS/MS often makes the operation process more tedious and complex. Although immunoassay realizes a simple and rapid detection procedure, there are still some restrictions, such as the preparation and purification of antigen and antibody, which constrain its application in conotoxin determination.

Electrochemical analysis (EA), known for its convenience and sensitivity, has shown broad application in the rapid detection of various substances. However, the poor selectivity of EA usually requires the assistance of other techniques as the recognition element [15]. Currently, the molecularly imprinted polymer (MIP) has been widely utilized in solid-phase extraction [16], chromatographic separation [17] and sensor fabrication [18]. MIP may sometimes be a substitute for biological receptors (e.g., enzyme, antibody, nucleic acid) due to its low cost, rapid preparation, physicochemical robustness and outstanding repeatability [19,20]. The employment of a combination of EA and MIP in the determination of drugs [21], biomolecules [22], environmental pollutants [23] and biotoxins [24,25] has emerged. Nevertheless, there is no report about using this combination method for conotoxin detection.

**Figure 1:**
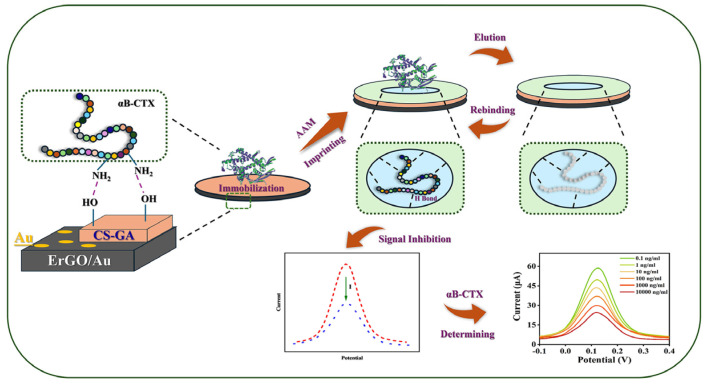
Fabrication of the rGO-Au modified MIP sensor for monitoring of the conotoxin αB-VxXXIVA.

Here, an MIP-based electrochemical sensor for αB-VxXXIVA was constructed on a screen-printed electrode (SPE) with the modification of electrochemically reduced graphene oxide, electrochemically reduced gold and polyacrylamide (PAM/rGO-Au/SPE) in this work (Figure 1). Considering the poor conductivity of MIP film and macromolecular conopeptides, graphene and gold were involved to amplify the electric signal of the sensor. In order to ensure the imprinting sites were situated on the surface of the electrode, chitosan and glutaraldehyde (CS-GA) were used as the cross-linking agents to immobilize the template molecule until it was removed by eluent. The αB-VxXXIVA could be recognized by MIP through hydrogen bonding, and the current peaks would be decreased inversely in relation to the concentration of αB-VxXXIVA.

## 2. Materials and Methods

### 2.1. Apparatus and Reagents

All electrochemical measurements were performed using a CHI 660E electrochemistry workstation from Shanghai CH instruments Co., Ltd. (Shanghai, China). The screen-printed electrode (SPE, TE200) was bought from Zensor R&D Co., Ltd. (Taiwan, China). Scanning electron microscopy (SEM) measurements were performed on Hitachi Regulus 8100 (Hitachi, Japan). Atomic Force Microscope (AFM) patterns were analyzed by Oxford Instruments MFP-3D (Oxford, UK).

Conotoxin αB-VxXXIVA was purchased from Yuanpeptide Biotech Co., Ltd. (Nanjing, China). Acrylamide (AAM), N,N′-methylene bisacrylamide (NNMBA), chitosan (CS), chloroauric acid and glutaraldehyde (GA) were purchased from Sigma Aldrich. Graphene oxide (GO) powder was purchased from Nanjing XFnano Materials Tech Co., Ltd. (Nanjing, China). Glutathione and L-cysteine were supplied by Shanghai Yuanye Bio-Technology Co., Ltd. (Shanghai, China).

### 2.2. Fabrication of the Molecularly Imprinted Polymer (MIP)-Based Sensor

An electrochemically reduced graphene oxide and gold (rGO-Au) composite was modified onto the surface of SPE through continuous electrodeposition. Firstly, 30 mg GO was dispersed in 30 mL ultrapure water under ultrasonication for 1 h. Then, 15 μL of the resulting GO suspension was dropped onto the working area of the SPE and was deposited under −1.2 V for 300 s. A HAuCl_4_ solution (15 μL, 5 mM) was then coated, and a potential of −0.4 V was applied to the electrode for another 300 s to finish the Au electrodeposition. The corresponding rGO-Au/SPE was then washed with distilled water and dried with nitrogen for further use.

To immobilize the macromolecular peptide, 5 μL CS (1%, *w*/*v*) and 5 μL GA (2.5%, *v*/*v*) solution were successively added to the rGO-Au/SPE, and the electrode was incubated under 4 °C for 2 h each. After washing and drying, CS-GA/rGO-Au/SPE was immersed in 5 mg/mL αB-VxXXIVA solution at 4 °C for 24 h and then washed and dried for further use.

The MIP was formed by the electropolymerization of AAM with NNMBA as a cross-linking agent. Then, 10 mg/mL AAM and 20 mg/mL NNMBA were mixed in phosphate-buffered saline (PBS, 0.1 M, pH = 7), and 15 μL of this prepolymer was dropped onto the αB-VxXXIVA/CS-GA/rGO-Au/SPE. The electro-polymerization was monitored by cyclic voltammetry (20 scans, −0.2 V to 1.2 V, 100 mV/s) to obtain PAM/αB-VxXXIVA/CS-GA/rGO-Au/SPE, which is MIP/rGO-Au/SPE. As shown in Figure S1, template molecules were removed by elution with acetic acid–methanol (40%, *v*/*v*) solution for 15 min [24,26]. The non-imprinted polymer (NIP) sensor was prepared in a similar manner in the absence of αB-VxXXIVA.

## 3. Results and Discussion

### 3.1. Electrochemical Properties of MIP-Based GO-Au Electrode

**Figure 2:**
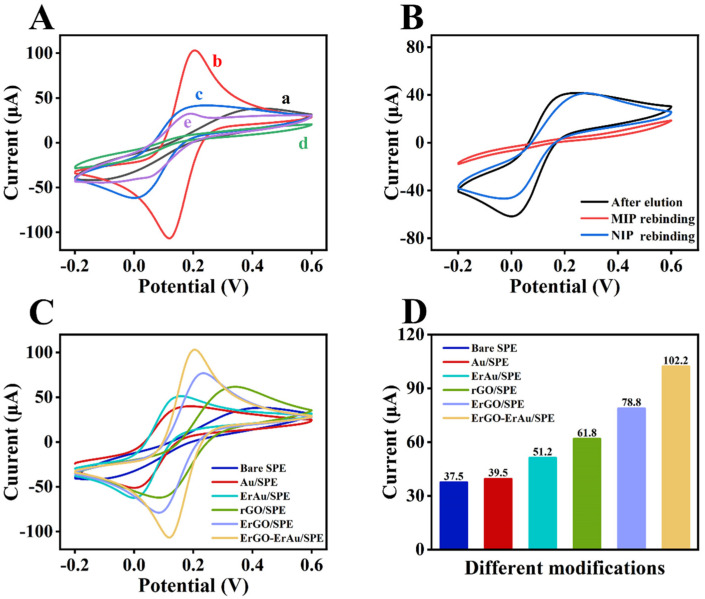
(**A**) CV of (a) bare SPE, (b) rGO-Au/SPE, (c) αB-VxXXIVA/CS-GA/rGO-Au/SPE, (d) MIP/rGO-Au/SPE and (e) MIP/rGO-Au/SPE (template removal); (**B**) CV of MIP and NIP before and after αB-CTX rebinding; (**C**) CV and (**D**) peak currents of SPE with different modifications of rGO and Au. CV curves were recorded in 5 mM [Fe(CN)_6_]^3−/4−^ in 0.1 M KCl range from −0.2 to 0.6 V (vs. Ag/AgCl).

Figure 2A displays the CV curves of bare SPE, rGO-Au/SPE, αB-VxXXIVA/CS-GA/rGO-Au/SPE, MIP/rGO-Au/SPE and MIP/rGO-Au/SPE (template removal) with ferricyanide solution as a redox probe. Bare SPE shows a moderate pair of peaks in the vicinity of 0.2 V, which is derived from the [Fe(CN)_6_] ^3−/4−^ (curve a). The modification of rGO-Au greatly enhances the conductivity of the sensor, which largely increases the peak current (curve b). After immobilization of the insulative peptide, the electron transport is hindered, and the peak current declines (curve c). The peak current further decreases to an extra low level through the polymerization of MIP due to the non-conducting PAM film (curve d). Template removal makes the surface of the sensor accessible again so that the redox reaction can take place smoothly, which can be verified by the recovery of the peak current (curve e) [27]. To determine whether the imprinted cavities can recognize the aimed molecule, the rebinding of αB-VxXXIVA was tested by eluted MIP and NIP sensors (Figure 2B). As illustrated, αB-VxXXIVA rebinding decreases the peak current for the occupancy of the capacity again, while the NIP sensor seems to have no response. The comparison can demonstrate that spatial cavity and hydrogen bonding are mainly attributed to specific recognition rather than non-specific physical adsorption.

Considering the influence of the insulative peptide and polymer on the electrical signal, different pathways of modifying conductive materials were investigated in order to find the best combination of rGO and Au. Figure 2C exhibits the CV curves of peak current affected by all five matches of modifiers, and the current values of each peak are displayed in Figure 2D. Compared to redox reduction, rGO and Au reduced by electrochemical reduction show relatively higher peak currents. Therefore, the rGO-Au composite was chosen to improve the conductivity of the sensor.

### 3.2. Morphology Characterization of MIP-Based rGO-Au Electrode

**Figure 3:**
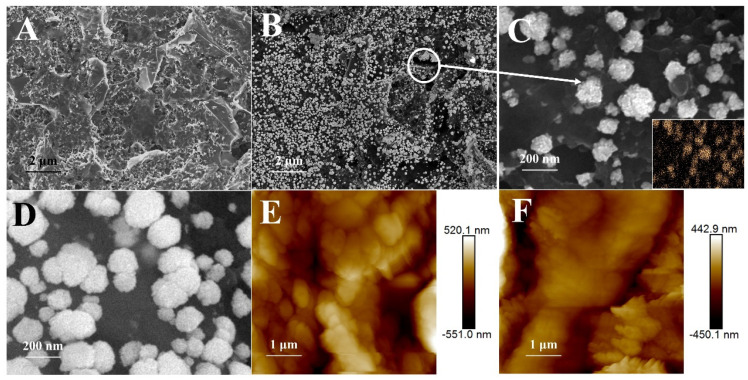
SEM images of (**A**) ErGO, (**B**) ErGO-ErAu and Au particles without (**C**) and with (**D**) MIP covered (inset: elemental mapping of Au on the electrode); AFM images of (**E**) MIP and (**F**) NIP.

A scanning electron microscope (SEM) was used to demonstrate the morphological characterizations of the fabricated sensor. rGO displayed a wrinkled and rough surface caused by the removal of oxygen-containing groups [28], which indicates that GO was reduced (Figure 3A). Au was then interspersed on the rGO layer through electrodeposition (Figure 3B). The diameter of Au particles is about 100–200 nm (Figure 3C). Figure 3D illustrates that αB-VxXXIVA was embedded in MIP during polymerization to form dense film outside Au particles so that the electron transport was extensively hindered. The structural characterizations were further confirmed by atomic force microscopy (AFM). The surface roughness of the MIP sensor and the NIP sensor is depicted in Figure 3E,F. The root-mean-square roughness (Rq) values of MIP and NIP are 154 nm and 112 nm, together with arithmetic-mean roughness (Ra) values of 120 nm and 83 nm, respectively. This can be attributed to the template molecules being entrapped in the MIP to make the surface of the sensor more rugged [29].

### 3.3. Sensing Performance of MIP-Based rGO-Au Electrode

**Figure 4:**
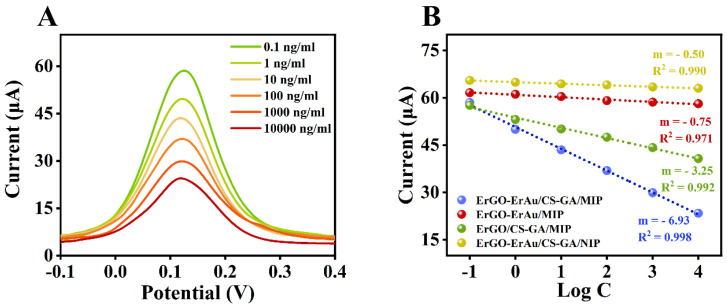
(**A**) DPV curves of MIP/rGO-Au/SPE in 5 mM [Fe(CN)_6_]^3−/4−^ in 0.1 M KCl range from −0.2 to 0.6 V (vs. Ag/AgCl) after incubation with solutions containing different concentrations of αB-VxXXIVA and (**B**) the calibration curves of MIP sensors with different fabrications and the NIP sensor.

To reveal the feasibility of the proposed MIP sensor in monitoring αB-VxXXIVA, several parameters, like pH of the polymerization electrolyte, number of electropolymerization cycles, target incubation time and ratio of functional monomer and template molecule, were optimized individually (Figure S2). Under the optimal conditions (pH = 7, 20 scans, 10 min incubation, 2:1 for functional monomer and template molecule), DPV was performed to measure the response with different concentrations of the target. As shown in Figure 4A, the peak currents present a progressive decrease with the concentration of αB-VxXXIVA going up (0.1 to 10,000 ng/mL). To analyze the data in this wide range, logarithm concentration was performed to descend the data range before linear calibration. The calibration curve derived from the data is illustrated in Figure 4B (blue line) with a function y = −6.93 log C + 50.76 (R^2^ = 0.998). The limit of detection (LOD) is calculated to be 0.014 ng/mL using the equation LOD = 3 S_b_/m (S_b_: blank standard deviation, m: slope of the calibration curve). In contrast, the NIP sensor shows a relatively weak response towards the aimed peptide with a low sensitivity (m = 0.50). The imprinted factor (IF) is 13.86, obtained by IF = m_MIP_/m_NIP_, which indicates that the specificity of the sensor towards αB-VxXXIVA is satisfactory. To further verify the feasibility of the MIP sensor in practical applications, the reproducibility, stability and selectivity were evaluated. According to the results, the fabricated sensor exhibits excellent reproducibility and stability (Figure S3), together with a splendid selectivity (Figure S3). The spatial cavity in the MIP sensor also reveals excellent selectivity, even in the presence of peptide analogs (Figure S4). All the operations can be finished within 15 min, and the method is pretreatment-free, while the assay time for traditional instrumental analysis is commonly over 40 min [24]. Thus, the proposed MIP sensor is confirmed to have potential for application in the rapid on-spot determination of αB-VxXXIVA.

Moreover, the contribution of peptide immobilization during the construction of MIP was also investigated by a response experiment (Figure S5). The synthesized MIP without target immobilization (red line) shows only negligible response and presents a similar low sensitivity with the NIP sensor (yellow line). Due to the lack of a cross-linking agent, macromolecular αB-VxXXIVA could probably cause the polymers to be fragile and ununiform or even lead to improper recognition, which makes the MIP sensor unusable. The MIP sensor containing Au (blue line) exhibits a higher sensitivity than that without Au participation (green line). This may be attributed to the CS-GA crosslinker, which immobilizes αB-VxXXIVA near the surface of the rGO-Au electrode. Consequently, the electrochemical signals greatly decrease. According to the results, the cross-linker is still necessary in the fabrication of MIP for macromolecules. Compared with other reported data in Table S1, the MIP-based electrochemical sensor reveals the advantages of fast operation, wide linear range and low limit of detection.

## 4. Conclusions

In summary, an electrochemical MIP/rGO-Au/SPE sensor was fabricated for monitoring conotoxin αB-VxXXIVA. The proposed sensor exhibits a broad linear range, low LOD, satisfactory specificity, and excellent reproducibility and stability, together with splendid selectivity. The amalgamation of electrochemistry and the MIP technique has been shown to offer a promising method for the rapid and sensitive determination of conotoxins. This methodology has the potential to be further extended to the monitoring of other macromolecular peptides.

## Data Availability

All data are included in this manuscript and supplementary information.

## Supplementary Materials

The following supporting information can be downloaded at: https://www.mdpi.com/article/10.3390/s25051408/s1, Figure S1: Selection of eluent with different volume fractions of HAc; Figure S2: Optimization of experimental factors: (A) pH of polymerization electrolyte, (B) number of electropolymerization cycles, (C) incubation time, (D) ratio of functional monomer and template molecule; Figure S3: (A) Reproducibility and (B) stability of the MIP sensor; Figure S4: Selectivity of the MIP sensor for different interfering species; Figure S5: (A) CV curves of the progress of CTX immobilization with or without cross-linker and (B) corresponding DPV curves of template elution; Table S1: Comparison of sensing performance toward αB-CTX. References [11,12,13,14,30] are cited in the supplementary materials.

## Abbreviations

The following abbreviations are used in this manuscript:

MIP	Molecularly imprinted polymer
NIP	Non-imprinted polymer
CTX	Conotoxin
αB-CTX	αB-VxXXIVA
GO	Graphene oxide
ErGO	Electrochemically reduced graphene oxide
ErAu	Electrochemically reduced gold
SPE	Screen-printed electrode
AAM	Acrylamide
PAM	Polyacrylamide
CS	Chitosan
GA	Glutaraldehyde
EA	Electrochemical analysis
