# Supplementary material for: Surface-Imprinted Acrylamide Polymer-Based Reduced Graphene–Gold Sensor in Rapid and Sensitive Electrochemical Determination of αB-Conotoxin
Source: Sensors (Basel). 2025 Feb 26;25(5):1408. doi: 10.3390/s25051408 (PMC11902586; doi:10.3390/s25051408)
Supplement: Supplementary file 1 [file sensors-25-01408-s001.zip › sensors-3451721-Supplementary.pdf]

Supporting Information

Surface imprinted acrylamide polymer based reduced-graphene-gold electrode in rapid and sensitive electrochemical determination of αB-conotoxin

1. The information of αB-VxXXIVA

The sequence of αB-VxXXIVA:

VRCLEKSGAQPNKLFRPPCCQKGPSFARHSRCVYYTQSRE

Molecular weight: 4629.29 Isoelectric point: 9.93

2. Selection of the eluent in MIP fabrication

Different eluents including water, ethanol, water-ethanol, sodium hydroxide solution, acetic acid-methanol solution and acetonitrile were evaluated for their template removal capacity. The MIP sensor was immersed in each eluent for 15 minutes and the acetic acid-methanol solution exhibited the best removal capacity for αB-VxXXIVA. This can be explained by the fact that the acid could break not only the hydrogen bonds between the template molecule and functional monomer, but also the covalent linkage by aldimine condensation.

However, an extremely strong acid could also destroy the MIP film or other modified materials as well. Therefore, the volume fraction of acetic acid was further optimized. The eluting performance and the corresponding sensing response to αB-VxXXIVA of different proportions of acetic acid-methanol solution are displayed in Figure S1. The ratio of 40% shows a satisfactory eluting performance together with the highest response value. Therefore, acetic acid-methanol (40%, v/v) was chosen as the eluent in this work.

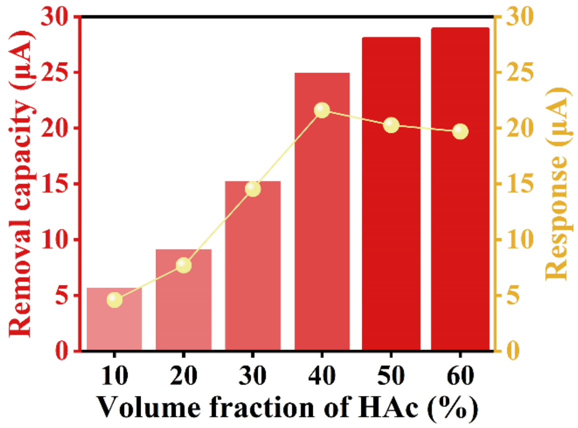

Figure S1 Selection of eluent with different volume fraction of HAc.

3. Optimization of experimental parameters

3.1 pH value of polymerization electrolyte

The pH of polymerization electrolyte is crucial to the fabrication of MIP. A pH that is too high or too low may interfere with the polymerization of acrylamide and hinder the formation of hydrogen bonds between AAM and αB-VxXXIVA.

Therefore, in this work, pH ranging from 4 to 9 were investigated in this work to find out the most suitable condition for MIP construction (Figure S2A). As illustrated in the Figure S2A, pH = 7 is the optimal pH pf the polymerization electrolyte, since it corresponds to the highest response to the target (ΔI).

3.2 Number of electropolymerization cycles

The Number of electropolymerization cycles determines the thickness of the MIP film, which is relevant for the stability of the MIP and the sensitivity of the sensor. In this work, the number of cycles were investigated from 5 to 35. The result in Figure S2B shows that ΔI increases rapidly from 5 to 20, while the increase becomes flat from 20 to 35. The sharp climbing of electric signal can be explained by the incomplete formation of the MIP at the beginning. As the number increases, the overly thick MIP film will hinder the electron transport and make the template molecule difficult to remove. Hence, a sweep of 20 cycles is chosen as the optimum number of electropolymerization cycles.

3.3 Optimization of incubation time

In order to avoid the false positive result caused by non-specific adsorption, an appropriate incubation time is essential. Figure S2C displays the effect of different incubation times. After 10 min, the ΔI becomes flat and exhibits a slight increase, indicating that the imprinted cavities have been filled up by αB-VxXXIVA after 10 min. Therefore, 10 min is chosen as the most suitable incubation time for target recognition.

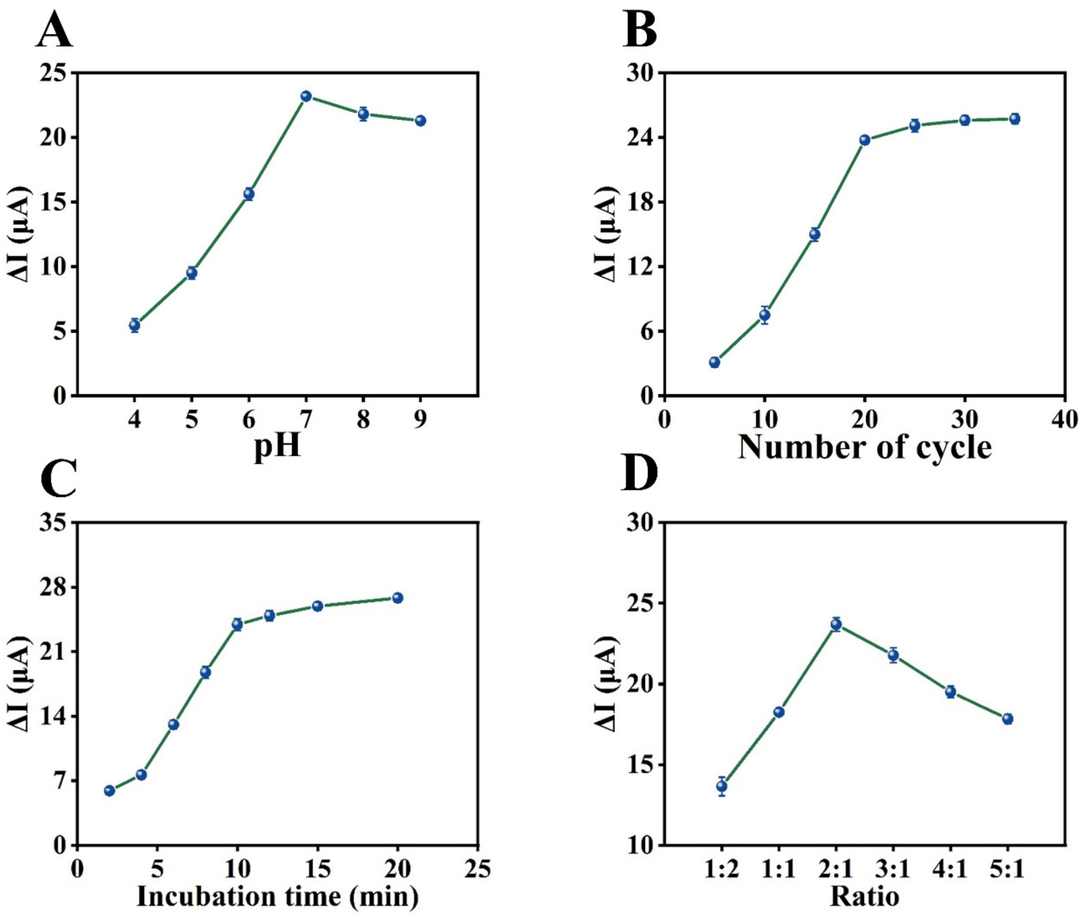

Figure S2 Optimization of experimental factors: **(A)** pH of polymerization electrolyte **(B)** number of electropolymerization cycles, **(C)** incubation time, **(D)** ratio of functional monomer and template molecule.

3.4 Ratio of functional monomer and template molecule

The ability of the MIP to recognize is largely dependent on the degree of matching between the imprinted cavities and the targeted molecules. An appropriate ratio of functional monomer and template molecule can better expose the active sites

and form a stable and uniform MIP film. To investigate the influence of the ratio on the sensing performance, a series of ratios of AAM and αB-VxXXIVA were applied (Figure S2D). It is obvious that 2:1 (AAM *vs.* αB-VxXXIVA, w/w) illustrates that the highest ΔI, which demonstrates such ratio is a considerable optimal proportion of reagents in polymerization.

4. The reproducibility, stability and selectivity of the MIP-based sensor

The practicability of the sensor was further verified by reproducibility, stability and selectivity experiments. The reproducibility was studied by recording the DPV peak currents of five consecutive assays. The signals show no obvious change between these measurements with a satisfactory RSD of 3.9% (Figure S4A). DPV is also used to assess the stability of the proposed sensor. The sensor was stored at 4 °C for 15 days and was tested by DPV in the presence of 1 μg/mL at different times. As illustrated in Figure S4B, the response remains 90% of the initial signal, indicating the possibility of long-term storage.

To confirm the feasibility of the MIP sensor in the application of the specific conotoxin monitoring, several interfering species including conotoxin ω-MVIIA, green fluorescent protein, glutathione, L-cysteine, glutamic acid and domoic acid were included to investigate the selectivity of the constructed recognition element. Figure S5 indicates that all the interferents have no obvious effect on the determination of αB-VxXXIVA and the percentage of signal from them is mostly less than 10%. The response of glutathione and L-cysteine is slightly higher than the rest. However, other sulphide-containing molecules, including the conotoxin ω-MVIIA and green fluorescent protein, show inconspicuous signals. This can be explained by the extreme structural mismatch between the species and the imprinted cavities. Due to the high specificity for the target, the MIP sensor shows a desirable selectivity and is competent in the detection of macromolecules with a complex matrix.

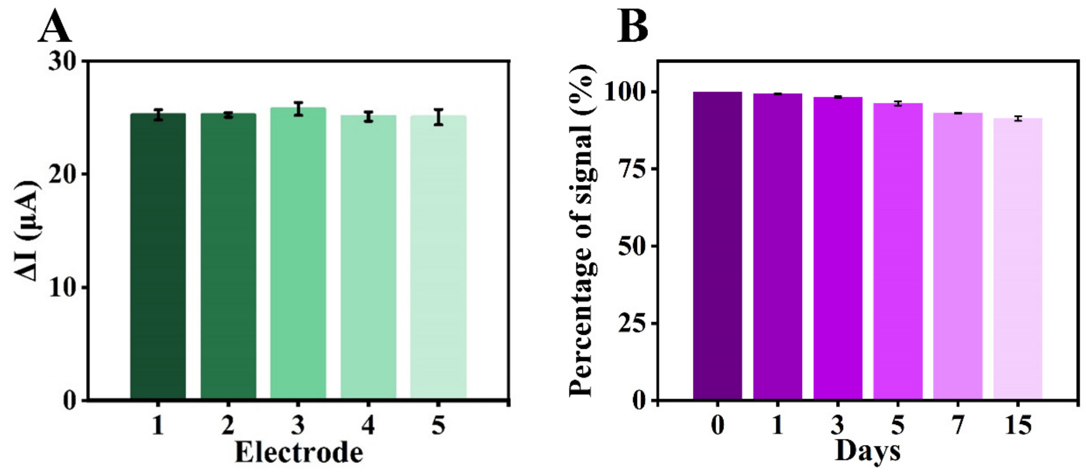

Figure S3 **(A)** Reproducibility and **(B)** stability of the MIP sensor. (n=3)

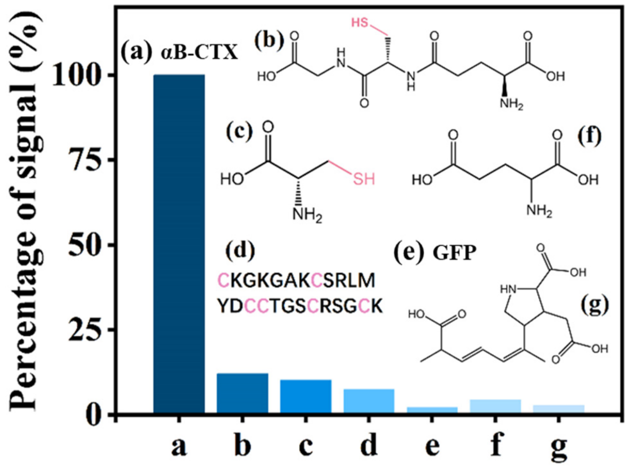

Figure S4 Selectivity of the MIP sensor for different interfering species: (a) αB-CTX, (b) glutathione, (c) L-cysteine, (d) conotoxin ω-MVIIA, (e) green fluorescent protein (GFP), (f) glutamic acid, (g) domoic acid.

5. Research of the immobilization of αB-VxXXIVA using cross-linking agent

To investigate the necessity of CS-GA, bonding, ErGO-ErAu/CS-GA/SPE and ErGO-ErAu/SPE were incubated with 5 mg/mL αB-VxXXIVA for 24 h, respectively. Figure S3A depicts the CV curves of these electrodes before and after target immobilization. The decrease in peak current is large for between ErGO-ErAu/SPE and ErGO-ErAu/CS-GA/CTX/SPE (about 40 μA), while it is only less than 20 μA for ErGO-ErAu/SPE and ErGO-ErAu/CTX/SPE. The DPV of each electrode before and after elution also shows that the signal recovery of ErGO-ErAu/CS-GA/SPE is almost

2-fold that of ErGO-ErAu/SPE (Figure S3B). These phenomena can be attributed to the cross-linker binding the target molecule through a specific recognition and hydrogen bonding. The immobilization by Au-S bond enhance the interaction of electrode and CS-GA, consequently leading to more recognition site in MIP sensor, which further confirms that the cross-linking agent is necessary for the fabrication of MIP for macromolecules.

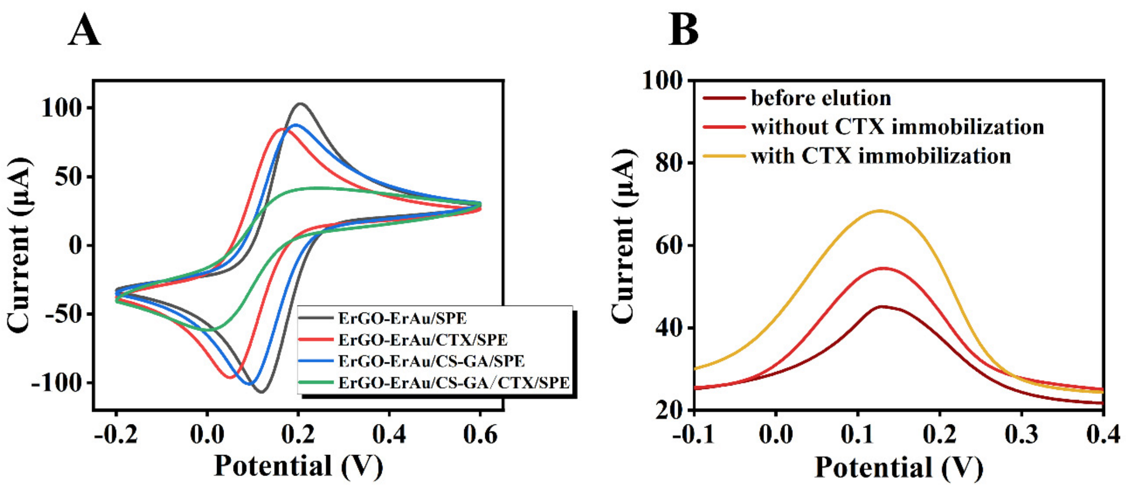

Figure S5 (A) CV curves of the progress of CTX immobilization with or without cross-linker and **(B)** corresponding DPV curves of template elution.

Table S1. Comparison of the sensing performance toward αB-CTX.

Method	Assay time (min)	Linear range (ng/mL)	LOD	Ref.
MALDI-TOF-MS	40	/	20 fmol	[1]
LC-ESI-MS/MS	>20	2-300	2 ng/mL	[2]
ELISA colloidal strip	<10	200-7220	1 μg/mL	[3]
Indirect competitive ELISA	/	117-3798	81 ng/mL	[4]
ELISA AuNPs-based strip	10	/	4 μg/mL	[5]
ELISA AuNFs-based strip	10	/	1.5 μg/mL	[5]
MIP electrochemistry		0.1-10000	0.014 ng/mL	This work

References

[1]. Junjian Fang, F. D., Na Wang, Kun He, Bingyu Liu, Shengming Wu, Ailing Li, and Xuemin Zhang, Rapid Detection of Conotoxin SO_3 in Serum Using Cu-Chelated Magnetic Beads Coupled with Matrix-Assisted Laser Desorption/Ionization Time-of-Flight Mass Spectrometry. *Journal of Analytical Toxicology* **2009,** 33, 272-277

[2]. Yu, S.; Yang, B.; Yan, L.; Dai, Q., Sensitive detection of α-conotoxin GI in human plasma using a solid-phase extraction column and LC-MS/MS. *Toxicon* **2019,** 158, S55-S56.

[3]. Wang, R.; Zhong, Y.; Wang, J.; Yang, H.; Yuan, J.; Wang, S., Development of an ic-ELISA and immunochromatographic strip based on IgG antibody for detection of ω-conotoxin MVIIA. *Journal of Hazardous Materials* **2019,** 378, 120510.

[4]. Tang, H.; Liu, H.; Gao, Y.; Chen, R.; Dong, M.; Ling, S.; Wang, R.; Wang, S., Detection of αB-Conotoxin VxXXIVA (αB-CTX) by ic-ELISA Based on an Epitope-Specific Monoclonal Antibody. *Toxins* **2022,** 14, (3), 166.

[5]. Tang, H.; Liu, H.; Gao, Y.; Chen, R.; Dong, M.; Ling, S.; Wang, R.; Wang, S., Development of Immunochromatographic Strip for Detection of αB-VxXXIVA-Conotoxin Based on 5E4 Monoclonal Antibody. *Toxins* **2022,** 14, (3), 191
